# Expediting the Management of Suspected Cauda Equina Syndrome (CES) in the Emergency Department Through Clinical Pathway Design at a District General Hospital: A Quality Improvement Project

**DOI:** 10.7759/cureus.32722

**Published:** 2022-12-20

**Authors:** Omer Nasim, Boulos Eskander, Zainab Rustam, Charalampos Pantelias, Robert Moverley

**Affiliations:** 1 Trauma and Orthopaedics, University Hospitals Dorset, Poole, GBR; 2 Emergency Department, Rehman Medical Institute, Peshawar, PAK

**Keywords:** cauda equina syndrome, clinical audit, quality improvement projects, district general hospital, patient-centred care

## Abstract

Background: Cauda equina syndrome (CES) is an uncommon condition that occurs due to compression of the terminal portion of the spinal cord. Early recognition and intervention in CES are crucial for an improved prognosis. Delayed diagnosis and action may lead to irreversible adverse effects, i.e., permanent disability, and in some circumstances can lead to litigation.

Aim: The aim of this quality improvement project (QIP) was to identify areas for improvement and expedite the management of suspected CES patients presenting to the hospital.

Material and methods: This was a retrospective study in which patients admitted to the Poole district hospital were analyzed in three groups with more than 50 patients in each subset group. The first group was audited from 1^st^ October 2020 to 27^th^ November 2020; a re-audit on the second group of patients was done from 1^st^ June 2021 to 16^th^ July 2021; the third group was re-audited from 1^st^ of January 2022 to 31^st^ of March 2022.

Results: There were a total of 168 patients in all audit groups, of whom 71% were female. The mean time from getting triaged to having an MRI improved from 13hrs 54mins to 10hrs 39mins. The total inpatient length of stay (LOS) of less than 24 hours was 28% in the first cycle and improved to 54.4% by the third cycle of the audit. Eight patients exhibited a diagnosis of cauda equina syndrome (CES) and were sent to the tertiary care center.

Conclusions: This quality improvement project identified delays in requesting the MRI for the diagnosis of CES and was addressed by ED booking the scans directly. This, in turn, reduced the length of stay in the hospital for patients who did not have cauda equina syndrome.

## Introduction

Cauda equina syndrome (CES) is the compression of the terminal portion of the spinal cord and roots of the spinal nerves, beginning at the first lumbar nerve root. Maxter "and" Barr described the condition first in English literature in 1934 [1]. Compression of these nerves leads to lower back pain, sciatica, saddle or genital sensory disturbances, and bladder, bowel, and sexual dysfunction [2,3].

The most common causes of CES are lumber disc herniation and spinal stenosis; other uncommon causes can include epidural hematoma, infections, ankylosing spondylitis, trauma, and neoplasms [4-8]. Due to varying combinations of symptoms, recognition of CES is often delayed. In a study by Shapiro et al., it was discovered that the average delay in diagnosing CES was nine days. Physician-related factors cause 83% of the delay [2].

Important historical elements to evaluate include a detailed history. It is important to inquire about changes in a patient’s bowel and bladder habits; difficulty voiding, incontinence, or loss of bowel control raises suspicion of CES. Physical examination includes examining sacral nerve roots. Pinprick sensation in the S2-S4 dermatomes, i.e., perianal region, posterior thigh, and perineum, must be checked. Decreased rectal tone, bulbocavernosus reflex, and anal squeeze test should be evaluated.

Postoperative spinal surgery patients and those on an anticoagulation regimen should raise suspicion of CES. Postoperative hematomas can lead to undiagnosed CES [9]. For patients who undergo elective spine surgery, routine use of chemical or mechanical thromboprophylaxis is not recommended. An emphasis on early and persistent mobilization has been placed [10].

A post-operative spine patient with back pain presents a unique and perplexing scenario to physicians. Post-operative back pain can be attributed to the expected postoperative course, but clinical suspicion must be high for CES if the back pain is persistent and urinary symptoms are present. A patient's anticoagulation regimen must be considered too. The preferred diagnostic modality for CES is magnetic resonance imaging (MRI) [11].

Early recognition and intervention in CES are crucial for an improved prognosis. Delayed diagnosis and action may lead to irreversible adverse effects and litigation consequences. The objective of this study was to evaluate the current practice of CES in our hospital from the patient's time of arrival to their management plan, try to determine factors that lead to delays in patient management, formulate a checklist for CES patients, and develop a hospital protocol.

## Materials and methods

**Figure 1:**
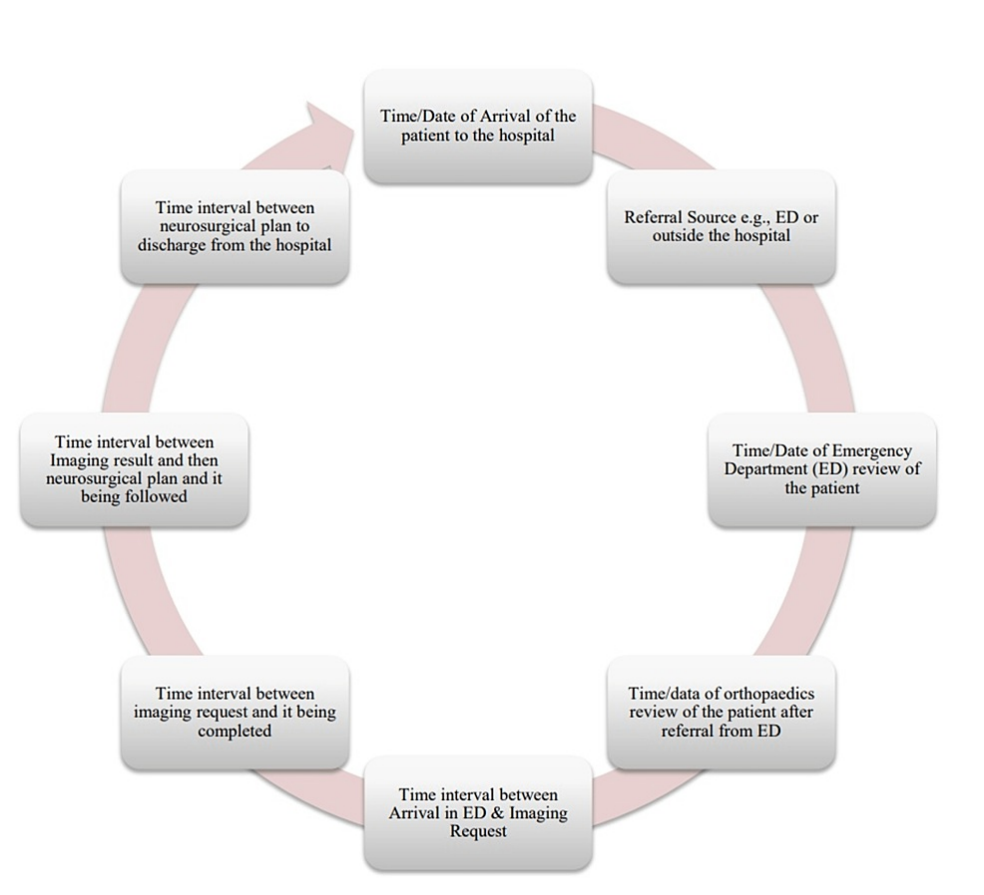
Time intervals recorded as per the data collection tool from the clinical journey of the patient through the hospital with suspected cauda equina syndrome ED: Emergency Department Image created by author Omer Nasim

This was a retrospective study in which patients admitted to the Poole district general hospital were analyzed in three audit cycles: the first two groups included 50 patients each, and the third cycle had 68 patients. A data collection tool was designed to include the patient's journey through the hospital. The patient's journey started at the emergency department, moved to radiological imaging, and then a discussion with the neurosurgical team to formulate a plan, followed by discharge from the hospital to either another hospital or home with conservative management. The time intervals were recorded as data points (Figure 1) to document the patient's clinical path.

The collection for these time intervals was sourced from the electronic patient record (EPR) used by these hospitals, and the software used by their emergency department (ED) to collect all time intervals/timestamps.

These time intervals were used and recorded into an Excel sheet (Microsoft Corp., Redmond, WA, USA) for further analysis. Formulas were used to calculate the mean time and range of time intervals for each of the data points, i.e., time intervals while the patient was being assessed at different points of their clinical journey through the hospital. The first group was audited from 1st October 2020 to 27th November 2020, the second group of patients from 1st June 2021 to 16th July 2021, and the third group from the 1st of January to 31st of March 2022.

Inclusion criteria were patients over the age of 18 who presented with complaints of lower back pain and neurological symptoms, and those who were referred as suspected CES patients to the orthopedic department. Those with vertebral fractures were excluded from the study, including pathological metastatic cord compression.

Analysis was completed with basic statistics of gender and age ranges, including the time intervals for each step of the clinical journey of the patient (as seen above in Figure 1). The approval for this audit was sought and granted by the University Hospitals Dorset's Audit Department (Reference # 5182). Informed consent was not required, as anonymized data were used.

## Results

The mean age remained relatively similar throughout the plan-do-study-act (PDSA) cycles; 43.3 (±16.94 ), 49.7 (±20.33), and 46.8 (±16.27), respectively (Table 1).

**Table 1:** Patient groups tabulated with age range and presentation with suspected cauda equina syndrome

Age groups (in years)	1^st^ Cycle (n=50)	2^nd^ Cycle (n=50)	3^rd^ Cycle (n=68)	Total (n=168)
<20	0	2	0	2
20-29	14	6	10	30
30-39	9	15	16	40
40-49	10	3	17	30
50-59	7	5	11	23
60-69	5	6	5	16
70-79	4	9	8	21
80-89	1	4	1	6
Grand Total	50	50	68	168

Table 2 includes the gender distribution through the three cycles of the audit, with 71% of females (n=120/168) being the total referrals for suspected CES to the orthopedics department. 

**Table 2:** Gender distribution through the cycles of the audits from the first to the third cycle

Gender	1st Cycle		2^nd^ cycle		3^rd^ cycle		Total	
	Frequency (n=50)	Percentage (%)	Frequency (n=50)	Percentage (%)	Frequency (n=68)	Percentage (%)	Frequency (n=168)	Percentage (%)
Males	16	32%	15	30%	17	25%	48	29%
Females	34	68%	35	70%	51	75%	120	71%

The majority of the referrals were from the hospital's emergency department (ED) but there were some from the general practitioner (GP) in the community and other specialty departments across the hospital (Table 3).

**Table 3:** Distribution of source of referrals with suspected cauda equina syndrome to the orthopedic department ED: Emergency Department, PGH: Poole General Hospital, GP: General Practitioner, RBH: Royal Bournemouth Hospital

Source of referral	1st Cycle		2^nd^ cycle		3^rd^ cycle		Total	
	Frequency (n=50)	Percentage (%)	Frequency (n=50)	Percentage (%)	Frequency (n=68)	Percentage (%)	Frequency (n=168)	Percentage (%)
ED @ PGH	26	52%	31	62%	62	91%	119	70.8%
GP	16	32%	14	28%	6	9%	36	21.4%
ED @ RBH	5	10%	5	10%	0	0%	10	6%
Rheumatology	2	4%	0	0%	0	0%	2	1.2%
Physiotherapy	1	2%	0	0%	0	0%	1	0.6%

Table 4 demonstrates the time intervals between each step of the patient's clinical journey (as seen above in Figure 1). The time intervals were documented and then the mean and the range were calculated for each instance of the clinical journey. The patients were initially reviewed by the ED, and then referred to the orthopedic department for further assessment, post which radiological imaging was requested. On the completion of imaging, the patient would be discussed with the spinal team at a tertiary care hospital for an appropriate plan. Then, a discharge plan would be formulated for the patient. The time intervals between each of these steps were calculated to devise a plan on how to address areas that could be improved. 

**Table 4:** Time frames of clinical interaction in the patient's clinical journey through the hospital ED: Emergency department, Ortho: Orthopaedic team, TOA: Time of arrival or the time a patient was seen by a triage nurse, CES: Cauda equina syndrome, LOS: Length of stay Action plan refers to the time at which a management plan is formulated by the tertiary care hospital, as documented on paper. *Patients had delayed discharge because of delayed physiotherapy review over weekends, having infections, or uncontrolled pain on pain team reviews. Patients with the highest in-patient LOS are related to complex discharge (d/c) plans such as a package of care (POC) or mental health issues.

Mean time (range)	1^st^ Cycle – mean time (range)	2^nd^ Cycle- mean time (range)	3^rd^ Cycle- mean time (range)
Patients reviewed initially in ED	Mean: 01:51:15	Mean: 02:32:12	Mean: 02:15:58
	Range: 00:07:00 – 04:09:00	Range: 00:20:00 – 11:39:00	Range: 00:06:00 - 10:36:00
Patients reviewed by ortho	Mean: 3:13:31	Mean: 4:40:23	Mean: 03:13:31
	Range: 0:15:00 – 9:59:00	Range: 0:18:00 – 14:42:00	Range 0:15:00 - 9:59:00
MRI request from time of arrival	Mean: 14:12:03	Mean: 7:32:10	Mean: 06:10:00
	Range: 1:26:00 – 138:39:00	Range: 00:29:00 – 22:47:00	Range: 04:00:00 - 17:47:30
MRI delay time (from order)	Mean: 7:48:19	Mean: 5:01:32	Mean: 4:29:08
	Range: 00:13:00 – 137:15:00	Range: 00:34:00 – 17:12:00	Range: 00:31:00 to 18:09:00
MRI completion time from TOA	Mean: 13:54:49	Mean: 12:24:00	Mean:10:39:00
	Range: 01:26:00 – 138:39:00	Range: 00:55:00 – 29:39:00	Range: 21:53:00 - 1:17:00
MRI results	CES diagnosed: 0%	CES diagnosed: 8%	CES diagnosed: 5.8%
Time of arrival to action plan	27:29:00	18:23:00	15:38:0
In-patient length of stay	Mean: 58:45:11	Mean: 51:51:50	Mean: 85:08:00
	Range: 07:25:00 – 184:04:00	Range: 03:34:00 – 365:51:00	Range 03:31:00 - 1179:00*

The time of arrival to having an action plan was the time interval from the arrival of the patient to the ED to having a definitive management plan according to their radiological imaging findings. These images were mostly discussed with the spinal team at a tertiary care hospital. This time interval showed a 33.1% improvement from the first cycle to the second cycle and a 43.1 % improvement comparing the third cycle with the first cycle, in terms of the reduction in the total-time interval (Table 5). 

**Table 5:** Tabulated figures for the length of stay in the hospital from the time of presentation at the ED to the time of discharge from the hospital

	1^st^ Cycle		2^nd^ Cycle		3^rd^ Cycle		Total	
	Frequency (n=50)	Percentage (%)	Frequency (n=50)	Percentage (%)	Frequency (n=68)	Percentage (%)	Frequency (n=168)	Percentage (%)
Stayed less than 24 hours	14	28%	18	36%	37	54.4%	69	41.1
24-48 hours	11	22%	21	42%	18	26.5%	50	29.8%
48-72 hours	6	12%	3	6%	5	7.4%	14	8.3%
>72 hours	19	38%	8	16%	8	11.7%	35	20.8%

Patients who were diagnosed with CES on MRI were referred by a GP and were sent to tertiary care urgently. The average time of getting the plan from the tertiary care was 4:06:45 (hours:minutes:seconds) and the average time of admission was 7:37:45 (hours:minutes:seconds) and this was in the second cycle. And another four patients showed CES in the third cycle and were sent via blue light emergency transfer to the tertiary care center. The rate of CES in the second cycle was 8% (n=4), and 5.8% (n=4) for the third cycle. The total number of patients requiring urgent surgical intervention at the tertiary care center was eight out of 168 (4.7%), which is higher than the national standard [12].

## Discussion

The purpose of this study was to assess the time it takes to admit a patient with back pain and reach a definitive diagnosis of CES. It is crucial to diagnose CES early to prevent permanent neurological dysfunction. A review of literature from the 1960s demonstrated it was not possible to recover from neurological dysfunction caused by CES [13]. However, further studies in the 1980s and early 2000s showed positive results, where patients who underwent a diskectomy for CES ended up regaining complete urinary function (17 out of 22), motor function (17 out of 22), and perianal sensation (13 of 15) [14,15].

The issues noted in our baseline audit were the time frames of reviewing a patient in the ED and the referral to orthopedics, which is the case at most district general hospitals due to the lack of a neurosurgical department, that were delaying the course of booking the MRI early for a definitive diagnosis. Most tertiary care hospitals will have 24-hour coverage for MRIs but at most district hospitals the service stops after six p.m. So, if there was a delay in reviewing the patient and the subsequent referral, the patient would not have the appropriate imaging and would be delayed till the next morning, prompting an overnight admission for observation.

**Figure 2:**
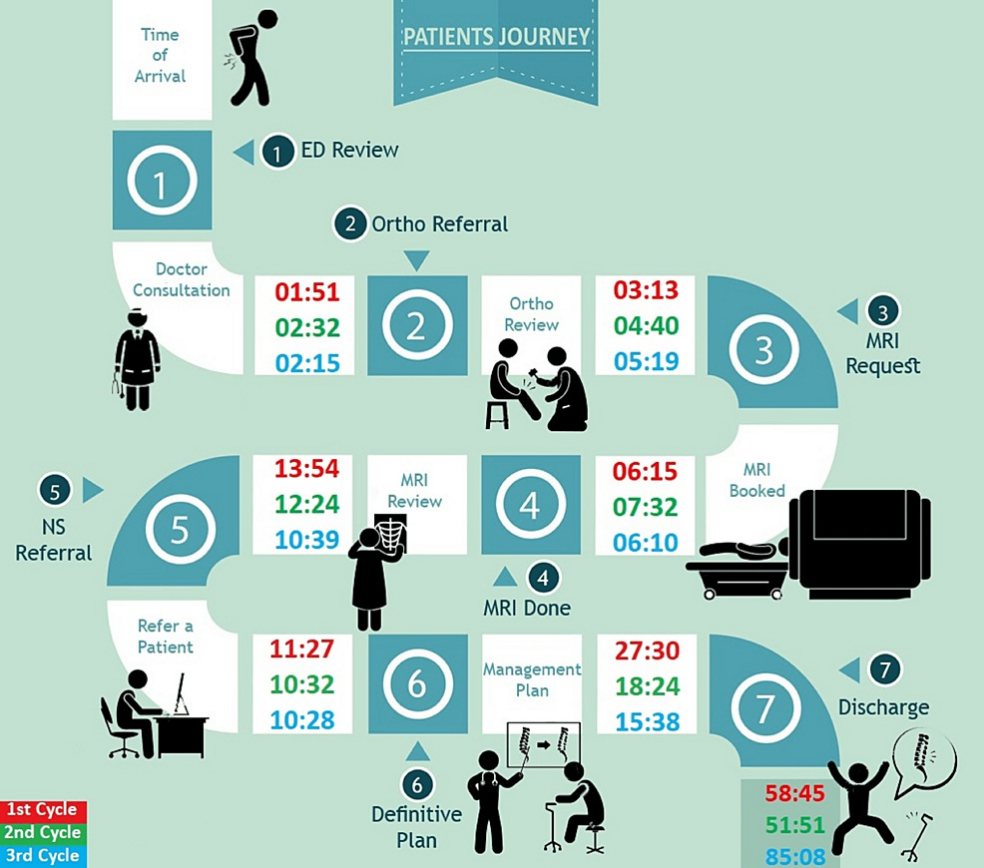
Time frame from the three audits on a patient's clinical journey through triage assessment to being discharged from the hospital with a definitive plan 1: Emergency department review; 2: Referral to the orthopedic department; 3: Request and booking of MRI; 4: Completion of MRI scan; 5: Neurosurgical referral via the electronic service; 6: Definitive plan (e.g., discharge, transfer to surgical intervention, local follow-up, conservative management); 7: Discharge from the hospital to home or another hospital in case of transfer for surgical intervention The format of the time intervals is hours:minutes Image designed by author Omer Nasim, and created by author Boulos Eskander

After establishing surgical management of CES as the definitive treatment, the time from diagnosis to intervention becomes essential. Several studies have shown that prolonged symptoms and delayed intervention lead to irreversible damage. Some studies recommend surgery be performed within 48 hours, but the exact timing is controversial [16,17]. Hence, we felt it was crucial to analyze the time it takes in our department to diagnose CES and start an intervention. Our findings revealed that out of the 50 referrals in our re-audit, four (8%) of our participants were diagnosed with CES, and the mean time it took to conduct their MRI scan was seven hours and 37 minutes from the time of their arrival (Figure 2). These times are well within the recommended 48-hour critical window. A study conducted in Hull reported 13% confirmed cases of CES when analyzing 250 referrals of suspected CES [18].

The diagnostic investigation of choice for CES is the MRI. A study found that the typical symptoms of CES such as urinary symptoms, back pain, and perineal symptoms, correlate poorly with the confirmed diagnosis of CES. Hence, an MRI is necessary to reach a definitive diagnosis of CES [19]. Another study assessing the reliability of clinical assessment to diagnose CES discovered that only 18.8% of the patients had CES [20]. Saddle sensory deficit had the highest positive predictive value when compared to other symptoms. However, it was established that no typical symptoms of CES have an absolute positive predictive value and it is necessary to perform an MRI to exclude the diagnosis of MRI [20].

Since evidence heavily supports the role of MRI in the definitive diagnosis of CES, a pathway should be established to allow access to MRI 24/7. This will reduce the timing from presentation to intervention, improve outcomes, and result in fewer litigation cases. It will also reduce the number of unnecessary referrals to the neurosurgical unit and hence decrease the burden and limit the wastage of resources [21,22].

PDSA 1: Sharing preliminary audit findings with Radiology and ED

The involved stakeholders are the ED and the radiology department, and the findings of the audit were presented to them. Specific timings were identified that could be improved by changes in the practice pertinent to the ED and radiology department, respectively. A consensus was reached that specific slots for imaging for patients suspected of having CES would be reserved by the radiology department, and the scans would be booked directly by the ED. And these scans would be prioritized for vetting by the radiologist. 

**Figure 3:**
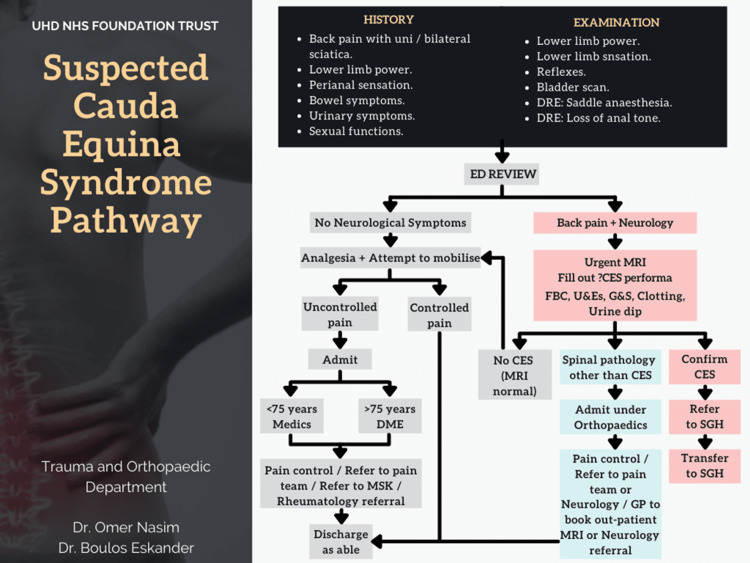
The proposed pathway for patients suspected with CES (1st PDSA cycle) CES: Cauda equina syndrome, PDSA: Plan-do-study-act, DRE: Digital rectal examination, ED: Emergency department, MRI: Magnetic resonance imaging, DME: Department of medicine for the elderly, SGH: Southampton General Hospital (tertiary care hospital) Image designed by author Omer Nasim, and created by author Boulos Eskander

Also, defined criteria and checklist were developed with red flags and strict protocol to document pre-void and post-void bladder scans. It was noted that the delay caused by the ED team by not booking the MRI immediately after reviewing a suspected case of CES was causing significant delays that could be improved if the ED would arrange for the scan before a referral to orthopedics. These delays were demonstrated to the relevant stakeholders via presentations and summary charts to the radiology department and ED. A provisional protocol pathway (Figure 3) was proposed for patients suspected of having CES. This was done so that the ED assessment would be sufficient for requesting an MRI on the suspicion of CES and in turn, would hasten the relevant radiological investigation.

PDSA 2: Posters and findings of improvement shared with stakeholders

The second PDSA cycle yielded better time intervals for the patient's journey through the ED and for having a definitive scan for diagnosis. The prompt booking of an MRI post-ED assessment of the patient reduced the MRI delay period from 7:48:19 to 5:01:32 (hours:minutes:seconds), which helped hasten the process of further management. A draft of the pathway that was being tested was made and shown to the stakeholders for improved MRI turnaround time and reduced length of stay (LOS) at the hospital.

PDSA 3: Finalizing the pathway for CES patients

The LOS in the hospital increased in the third cycle of the audit. Through the cycles of the audit after the due presentation and formulation of the protocol pathway (as seen above in Figure 3), we were seeing more patients stay in the hospital for less than 24 hours, a faster clinical decision from the time of arrival at the ED, and radiological imaging that hastened their clinical journey.

To establish a set pathway for these patients, a proposed pathway was initiated with distribution to all the ED personnel and was also shared among the orthopedic department doctors for clarity on how to proceed with these patients.

Recommendations

Establishing a pathway that deals with CES immediately can have a significant impact on the financial burden caused by the majority of the cases. An outpatient pathway could reduce the impact on hospital resources such as manpower and diagnostics caused by inpatient stays without intervention due to delays in the booking of scans. This will also lead to better patient satisfaction as reduced hospital stays and early discharge can prevent the frustrations and anxieties of prolonged hospital stays.

Service for such a subset of patients could be improved with a spinal surgeon's clinic appointment after their presentation to the hospital with a scan that does not show CES but has some spinal pathology. In some hospitals, spinal service is not present, such as in district hospitals. This lack can overwhelm a region's local spinal service, but this could be addressed by the regional spinal service via telephone or virtual consultation. This approach could settle patients' anxieties and answer their questions regarding their symptoms.

## Conclusions

This quality improvement project identified delays in diagnosing CES, which can cause irreversible damage; hence, early diagnosis is important. Time intervals and their analysis showed how to reduce the time to have a definitive plan for these patients in less than 24 hours. The majority of the patients were found not to have CES on the gold standard investigation, but the ones that required urgent surgical decompression were higher than the national standard. In conclusion, the ED must book radiological imaging early after review without delays or referrals to orthopedics to reduce the inpatient hospital stay and the time taken to make a definitive plan.

## References

[1] Mixter WJ, Barr JS (1934). Rupture of the intervertebral disc with involvement of the spinal canal. N Engl J Med.

[2] Shapiro S (1993). Cauda equina syndrome secondary to lumbar disc herniation. Neurosurgery.

[3] Ahn UM, Ahn NU, Buchowski JM, Garrett ES, Sieber AN, Kostuik JP (2000). Cauda equina syndrome secondary to lumbar disc herniation: a meta-analysis of surgical outcomes. Spine (Phila Pa 1976).

[4] Kebaish KM, Awad JN (2004). Spinal epidural hematoma causing acute cauda equina syndrome. Neurosurg Focus.

[5] Cohen DB (2004). Infectious origins of cauda equina syndrome. Neurosurg Focus.

[6] Bagley CA, Gokaslan ZL (2004). Cauda equina syndrome caused by primary and metastatic neoplasms. Neurosurg Focus.

[7] Rubenstein DJ, Alvarez O, Ghelman B, Marchisello P (1989). Cauda equina syndrome complicating ankylosing spondylitis: MR features. J Comput Assist Tomogr.

[8] Harrop JS, Hunt GE Jr, Vaccaro AR (2004). Conus medullaris and cauda equina syndrome as a result of traumatic injuries: management principles. Neurosurg Focus.

[9] Geerts WH, Heit JA, Clagett GP, Pineo GF, Colwell CW, Anderson FA Jr, Wheeler HB (2001). Prevention of venous thromboembolism. Chest.

[10] Hirsh J, Guyatt G, Albers GW, Schünemann HJ (2004). Proceedings of the seventh ACCP conference on antithrombotic and thrombolytic therapy: evidence-based guidelines. Chest.

[11] Coscia M, Leipzig T, Cooper D (1994). Acute cauda equina syndrome. Diagnostic advantage of MRI. Spine (Phila Pa 1976).

[12] (2022). Organising quality and effective spinal services for patients: a report for local health communities by the Spinal Taskforce: Department of Health - Publications. January.

[13] Schaeffer HR (1966). Cauda equina compression resulting from massive lumbar disc extrusion. Aust N Z J Surg.

[14] Kostuik JP, Harrington I, Alexander D, Rand W, Evans D (1986). Cauda equina syndrome and lumbar disc herniation. J Bone Joint Surg Am.

[15] Buchner M, Schiltenwolf M (2002). Cauda equina syndrome caused by intervertebral lumbar disk prolapse: mid-term results of 22 patients and literature review. Orthopedics.

[16] Tarulli AW (2015). Disorders of the cauda equina. Continuum (Minneap Minn).

[17] Chau AM, Xu LL, Pelzer NR, Gragnaniello C (2014). Timing of surgical intervention in cauda equina syndrome: a systematic critical review. World Neurosurg.

[18] Hussain MM, Razak AA, Hassan SS, Choudhari KA, Spink GM (2018). Time to implement a national referral pathway for suspected cauda equina syndrome: review and outcome of 250 referrals. Br J Neurosurg.

[19] Bell DA, Collie D, Statham PF (2007). Cauda equina syndrome: what is the correlation between clinical assessment and MRI scanning?. Br J Neurosurg.

[20] Balasubramanian K, Kalsi P, Greenough CG, Kuskoor Seetharam MP (2010). Reliability of clinical assessment in diagnosing cauda equina syndrome. Br J Neurosurg.

[21] British Association of Spine Surgeons (2022). Standards of Care for Investigation and Management of Cauda Equina Syndrome. Society of British Neurological Surgeries.

[22] Germon T, Ahuja S, Casey AT, Todd NV, Rai A (2015). British Association of Spine Surgeons standards of care for cauda equina syndrome. Spine J.

